# Machine learning analysis of non-marital sexual violence in India

**DOI:** 10.1016/j.eclinm.2021.101046

**Published:** 2021-08-01

**Authors:** Anita Raj, Nabamallika Dehingia, Abhishek Singh, Julian McAuley, Lotus McDougal

**Affiliations:** a Center on Gender Equity and Health, Department of Medicine, University of California San Diego, San Diego, CA, USA; b Joint Doctoral Program-Public Health, San Diego State University and University of California San Diego, San Diego, CA, USA; c International Institute of Population Sciences, Mumbai, India; d Department of Computer Science, School of Engineering, University of California San Diego, San Diego, CA, USA

**Keywords:** Gender-based violence, Sexual violence, Machine learning, India

## Abstract

**Background:**

Machine learning techniques can explore low prevalence data to offer insight into identification of factors associated with non-marital sexual violence (NMSV). NMSV in India is a health and human rights concern that disproportionately affects adolescents, is under-reported, and not well understood or addressed in the country.

**Methods:**

We applied machine learning methods to retrospective cross-sectional data from India's nationally-representative National Family Health Survey 4, a demographic and health study conducted in 2015–16, which offers 4000+ variables as potential independent variables. We used Least Absolute Shrinkage and Selection Operator (lasso) or L-1 regularized logistic regression models as well as L-2 regularized logistic regression or ridge models; we conducted an iterative thematic analysis (ITA) of variables generated from a series of regularized models.

**Findings:**

Thematic analysis of regularized models highlight that past exposure to violence was most predictive of NMSV, followed by geography, sexual behavior, and poor sexual and reproductive health knowledge. After these, indicators largely related to resources and autonomy (e.g., access to health services, and income generating) were associated with NMSV. Exploratory analysis with the subsample of never married adolescents 15–19 years old, a population with higher representation of recent NMSV, further emphasized the role of wealth and mobility as key correlates of NMSV, along with poor HIV knowledge, tobacco use, higher fertility preferences, and attitudes accepting of marital violence.

**Interpretation:**

Findings indicate the validity of machine learning with iterative theme analysis (ITA) to identify factors associated with violence. Findings were consistent with prior work demonstrating associations between NMSV and other violence experiences, but also showed novel correlates such as lower SRH knowledge and service utilization and, for girls, norms and preferences suggesting more restrictive gender norms. Sexual and reproductive health, gender equity and safety focused interventions are important for addressing NMSV in India, particularly for adolescents.

**Research in context**

**Evidence before this study**

Machine learning allows for better consideration of a wider array of potential risk factors by employing algorithms to parse data, running through multiple iterations of variables in the dataset to learn the optimum model for explaining outcomes. Existing literature highlights machine learning as an analytic technique adapted for use in public health, but minimally applied in the area of gender-based violence. This study tests application of machine learning to understand non-marital sexual violence (NMSV) among women in India, an outcome that has received less attention from quantitative research, in part due to low reporting.

**Added value of this study**

Findings confirm existing evidence with regards to sociodemographic factors being associated with NMSV, and extends prior research by demonstrating that women with economic agency (e.g., income generation, control over income) and health care knowledge and access are more likely to report NMSV, as are adolescent girls with high mobility and non-residence with family. Findings from this work belie assumptions that the most socially vulnerable girls are at higher odds for sexual violence.

**Implications of all the available evidence**

Machine learning techniques offer a useful research tool to understand gender-based violence in contexts with low reporting. We need more focus on NMSV prevention efforts to address male NMSV norms and to support female safety in mobility.

Alt-text: Unlabelled box

## 1 Introduction

Population-based survey data is a cornerstone of public health research, allowing for examination of social and behavioural health indicators as well as clinical indicators for a more comprehensive analysis of public health risk factors and outcomes. These commonly offer wider data sets, i.e., data with many potentially relevant variables for each subject due to the broad health areas covered in such surveys [1]. For example, the Demographic and Health Survey (DHS), is a household surveillance system conducted across 90 countries and capturing a wide array of demographic and health indicators, including maternal and child nutrition, household water sources, marital violence, and education and employment data [2]. These are typically analysed via standard hypothesis-driven epidemiologic and demographic analyses, narrowing the number of potential correlates tested to reduce vulnerability to the problem of multiple comparisons, which can yield spurious significant findings. However, there is increasing recognition that such approaches limit consideration of the full surveillance data set, sparking interest and use of machine learning in public health to allow for broader consideration of more potential correlates, using an exploratory rather than hypothesis driven approach [3], [4], [5], [6], [7]. Machine learning is particularly useful for less understood social and health phenomena within a particular context or population, where exploratory analysis may support improved understanding of these.

Machine learning allows for better consideration of a wider array of potential risk factors by employing algorithms to parse data, running through multiple iterations of variables in the dataset to learn the optimum model for explaining the outcome of focus [1,3,8]. Machine learning algorithms are best engaged for very large datasets, such as national DHS data. To date, the approach has received limited use on issues of gender-based violence or gender inequalities. However, a recent study from our team using DHS data from India, known as the National Family and Health Survey in this country context, was used to understand child marriage [9]. Results revealed novel correlates of this outcome -non-use of public health nutritional services, a finding that corresponded to prior data on associations between child marriage and poor nutritional health in India [10,11]. Further, the findings reinforced prior research on the topic with this same data set but using traditional epidemiologic analysis, showing that rural, poorer, and less educated females were more likely to have married as minors, indicating validity of this approach for analysis [9]. The techniques used in this paper included an iterative thematic analysis (ITA) of variables and iterative regularized models to expand conceptual understanding of identified correlates of child marriage, an approach developed by this research team. The study then used neural networks to assess via a single model, the variables that best explaine the outcome, further offering cross-validation of findings from our ITA approach for a more robust analysis [9]. Hence, the machine learning approach with ITA demonstrated validity and provided novel correlates, suggesting the value of this approach for understanding gender inequalities.

An additional area requiring greater understanding in India is that of non-marital sexual violence (NMSV). Sexual violence is a health and human rights issue that affects more than 20 million women aged 15–49 years in India, or 6% of the female population in this age range, most often first occurring in adolescence or young adulthood [12,13]. National data indicate that most sexual violence occurs in marriage; among women in India reporting sexual violence, 83% report their current husband and 9% percent report a former husband as the perpetrators [12,13]. NMSV is less understood, in part due to gross under reporting due to stigmatization of victims of sexual assault and pre-marital sex in the country [14]. Inadequate analysis on this topic is in strong contrast to the extensive media coverage and public outcry on the topic. Research on sexual violence across types of perpetrators, including husbands, suggests that there is greater vulnerability for such assaults among women and girls who are poorer, rural, less educated, and those in Scheduled Tribes/Scheduled Castes [12]. However, more recent evidence, focused on unmarried adolescents reporting NMSV using these same data, reveal a different picture, with urban, wealthier, and more mobile girls having greater odds of having experienced NMSV [15].

While more research is clearly needed to elucidate these findings, low reports of NMSV impede analysis, even in the very large national data set available from India [12]. To that end, machine learning may be useful to maximize analysis of available data on the topic to increase our understanding.

In this study, we used machine learning to identify correlates of NMSV, testing this previously validated approach inclusive of regularized models, iterative regularized models with thematic generation, and neural networks, and using these models to explore and identify previously unrecognized correlates of this outcome. As with the prior work on child marriage, analyses used survey data from a very large nationally representative sample of women aged 15–49 years in India. We additionally explored NMSV for the subsample of unmarried adolescent girls 15–19, given the important associations in this sample identified using traditional epidemiologic methods [15]. Focus on the adolescent subsample not only provides information specific to younger unmarried girls, a population more vulnerable to NMSV, it allows for additional validation of the approach. These findings can guide application of machine learning methods to understand issues of gender-based violence via use of large-scale survey data, and can provide insights for NMSV prevention and intervention in India.

## 2 Methods

Data were drawn from the National Family Health Survey (NFHS-4), a nationally-representative, household-based survey, conducted during 2015–16 in India. NFHS-4 used the Census of India 2011 sampling-frame of 640 districts from 29 states and 7 Union Territories (UTs) of India. A total of 628,900 households were selected for the sample and approached for recruitment. Of the 616,346 households that were occupied, 601,509 agreed to interview, for a response rate of 98%. For these households, 723,875 eligible women age 15–49 were identified for individual women's interviews, and 699,686 women agreed to and participated in the interview, for a response rate of 97%. A subsample of 15% of households were selected for state representation, for which women were also asked about experience of sexual violence. These households provided the subsample for this study. The detailed sampling strategy for this survey has been described elsewhere [12].

NFHS-4 interviewed women of age 15–49 years old on aspects related to their health, with a focus on maternal, child and reproductive health, access to health services and health providers, and household characteristics related to social and economic status. A sub-sample of women was also interviewed on their experiences of violence, as well as dimensions related to their agency and empowerment. The current analysis used this sample of women who responded to questions related to experiences of violence (*N*=79,279), a subsample also designed to reflect state and national representation. This subsample included both ever married and never married women to ensure generalizability in India, though our outcome was limited to NMSV (excluding marital sexual violence. Inclusion of ever-married women is important as marriage is largely ubiquitous in India; 78% of our sample of 15-49 year olds were currently or previously married.

The complete analysis was also repeated for a sub-sample of these women aged 15 to 19 years old, and who had not ever been married at the time of interview (*N*=8,007). Although 17% of adolescent girls in our total sample were married, we excluded these to obtain more insight into NMSV, which disproportionately affects adolescent girls and is most commonly the age at which first experiences of sexual violence occur [12,14]. We were also able to validate our findings for this adolescent subsample against prior epidemiologic study focused on unmarried girls [15]. The study was exempted from ethical approval from the IRB at UCSD (USA) as it is based on publically available de-identified data.

### 2.1 Measures

We defined our outcome of interest, NMSV, using the survey item on whether at any time in their life, as a child or as an adult, anyone other than their husband forced the respondent to have sexual intercourse or perform any other sexual acts when they did not want to. If a respondent indicated no sexual violence or if they indicate husband as perpetrator and no other perpetrator, we categorized them as “no” on NMSV. All others reporting sexual violence were categorized as “yes” on NMSV.

The analysis included all variables from the NFHS-4 dataset, as the study aimed to explore potential correlates of NMSV from a large dataset with information on diverse aspects related to the women. This large set of independent variables were pre-processed prior to inclusion in the statistical models, using an approach outlined in Appendix A. All categorical variables were one-hot encoded, whereby categorical variables are converted into multiple binary forms [16]. After the pre-processing strategy, in total, each woman was represented as a set of over 6,500 variables that summarized her characteristics.

### 2.2 Statistical analysis

As noted above, the analytical method followed in this study was validated previously using the NFHS-4 dataset [17]. Similar to this prior study, we used three supervised machine learning models: a) an L-1 regularized logistic regression model, or Least Absolute Shrinkage and Selection Operator (lasso), b) an L-2 regularized logistic regression model, or ridge, and c) a neural network model, all of which have been extensively used in prior applied research [7,[18], [19], [20]]. The original study which validated the use of these three machine learning tasks found them to outperform traditional regression models, as well as other models including random forest model [17].

As with a typical supervised machine learning task, we first split the dataset into training and test data, with 20% of the data randomly assigned as test dataset. (See Appendix A for details.)

We then conducted our models and evaluated them using two metrics: Balanced Error Rate (BER), and Area Under Curve of Receiver Operating Characteristic (AUC). BER is the average of errors for positive and negative classification of the outcome feature. It is calculated as [1-0.5*(True Positive Rate + True Negative Rate)]. AUC is another metric indicating accuracy of the models; it is a plot of the test true-positive rate (y-axis) against the corresponding false-positive rate (x-axis); i.e., sensitivity against specificity [21]. With highly unbalanced datasets such as ours, where prevalence of NMSV is very low, AUC and BER take into account both true positive and true negative rates, and thus provide reliable metrics for model evaluation.

Broadly, our analysis consisted of two strategies: 1) lasso followed by ridge model for iterative categorization of themes, i.e., iterative thematic analysis (ITA), and 2) lasso followed by neural network model. We identified key correlates of NMSV from the two independent strategies.

We conducted all analyses using Python with necessary libraries (pandas, scipy, keras, numpy, sklearn, tensorflow) to develop the predictive algorithms.

### 2.3 Lasso followed by ridge model for iterative thematic analysis (ITA)

Lasso regression and ridge regression models, are both forms of regularized regression models. These forms of regularization impose a penalty on the size of logistic regression coefficients by trying to shrink them towards zero [22]. Regularized estimators are thus restricted maximum likelihood estimators (MLE), since they maximize the likelihood function subject to restrictions on the logistic regression parameters. Thus, in datasets involving high dimensionality with a large number of variables, issues related to multicollinearity, and over-fitting can be overcome by regularizing the regression models.

Since our analysis included a wide dataset with a large number of variables, we first trained a regularized regression model that is often used for variables selection: lasso. Lasso models are used for variable selection and shrinkage, as they can force some coefficient estimates to be exactly equal to zero, [22,23] allowing identification of variables that are least correlated with our outcome of interest. Following lasso, we implemented a ridge regression model (with the set of variables that had coefficient values higher than zero from the lasso model). Ridge regression models shrink the coefficient values towards zero, but never to an absolute zero, making it suitable for use when trying to identify coefficient values of variables that have some known potential relationships. We implemented lasso prior to the ridge regression models, to ensure use of parsimonious models for ridge; lasso allowed for reduction of noise in the ridge regression models since we were dealing with a “wide” dataset, i.e. a dataset with large number of variables. The results from the ridge regression were ranked in terms of their coefficient values. The variables with coefficient value higher than the knee point or point of maximum curvature of the coefficient curve were selected for further analysis and interpretation. We used the mathematical definition of curvature for a continuous variable as the basis of the knee-point definition for our analysis [24]. These selected variables with coefficient values higher than the knee point were then retained for further qualitative inspection.

### 2.4 Iterative thematic analysis (ITA)

We implemented these two models, lasso followed by ridge, using a strategy of iterative thematic analysis (ITA), developed and used in prior machine learning research with this same dataset [9]. This strategy was based on the fundamentals of qualitative coding of information, where domain experts independently review text to generate and code relevant themes [25]. Two experts independently coded the text, inter-reliability was tested (>90%), and then coders met to reach consensus for any codes in dispute. A group of variables was identified as a theme when the number of variables within was at least 5% of the total number of identified variables above the knee point of the coefficient curve. A single variable could be included in multiple themes.

Once the themes from the first model were generated and coded, the features attached to the theme which had the highest variance, or highest coefficient value, were identified. These features were dropped, and the machine learning models of lasso and ridge were fit again, followed by the thematic categorization of variables by the coders. This ITA process of using machine learning models and qualitative coding was carried out until no new themes were identified for at least three consecutive iterations, found no new variables in a given iteration, or the AUC was less than 65%, reliant on the research question [9,26]. For our analysis, the first iteration where all variables were included in the model had an AUC of 80%.

### 2.5 Lasso followed by neural network model

In addition to the regularized logistic regression models, we also used artificial neural network models to account for non-linear relationships among the predictors. Prior to implementing a neural network model, we used lasso, again, to eliminate noise from our “wide” dataset and allow for a parsimonious input dataset with reduced variables for the neural network model.

We used feed - forward neural networks, where the input travels in one direction. Between the input and output nodes, are the hidden layers. The layers define the successive linking of inputs and outputs. The higher the number of hidden units, the higher is the functional complexity of the equation relating input to outcome. The value of each hidden unit was calculated by summing the product of the input units with their associated weights, and applying a non-linear activation function to this summation. The activation functions are simple transformations to ensure that the predicted output falls in the required range. In this study, the output required a binomial response, so we used *tanh* function to perform this transformation. *Tanh* is often used for classification algorithms. The model used batch normalization, with 100 epochs and a batch size of 100.

### 2.6 Role of the Funding Source

The funders had no role in study design, data analysis, generation of the paper, and the decision to submit this paper for publication. All authors had access to the data and decided to submit this work for publication without input from the funder.

## 3 Results

This analysis included data from 79,279 eligible women, around 1% of whom had experienced NMSV in their lifetime (Table 1). On average, sampled women were 30 years old, and had 7 years of education; nearly two-thirds (65%) lived in rural areas. We found higher prevalence of having never married (30% vs 22%), being in the poorest quintile (20% vs 17%), urban residence (41% vs. 36%), and scheduled tribe/scheduled casted (SC/ST, i.e., lower caste) for those with a history of NMSV compared with the sample as a whole. Perpetrators of NMSV included current/former boyfriends and former husbands (45%), relatives (24%), friend or acquaintance (6%), family friends (7%), strangers (4%), brother or step-brother (3%) and father or step-father (3%). About 32% of those who experienced NMSV ever disclosed their abuse, formally or informally; most of these engaged in informal disclosure (e.g., friend or family).

**Table 1:** Sample characteristics of participants.

	Total sample (15–49 years old) (*N* = 79,279)	Sub-sample of women (15–49 years) who experienced NMSV (*n* = 847)	Sub-sample of women (15–49 years) who did not experience NMSV (*n* = 78,432)	*p*-value	Sub-sample of never married adolescent girls (15–19 years) (*n*=8,007)	Sub-sample of women (15–49 years) who experienced NMSV (*n* = 120)	Sub-sample of women (15–19 years) who did not experience NMSV (*n* = 7,887)	*p*-value
Characteristics	%/Mean (Std. err)	%/Mean (Std. err)	%/Mean (Std. err)			%/Mean (Std. err)		
Current age	30.04 (0.06)	29.05 (0.68)	30.04 (0.06)	0.818	16.72 (0.02)	16.74 (0.25)	16.72 (0.02)	0.947
Years of schooling	7.01 (0.03)	7.12 (0.32)	7.01 (0.03)	0.564	9.22 (0.05)	9.27 (0.36)	9.22 (0.05)	0.908
Marital status								
Never married	22.35%	30.37%	22.22%	0.000	100%	100%	100%	-
Currently married	73.35%	62.66%	73.53%		-	-	-	
Widowed/Divorced/Separated	4.30%	6.97%	4.25%		-	-	-	
Wealth index status:								
Poorest	16.50%	20.41%	16.44%	0.061	18.20%	15.92%	18.19%	0.106
Poorer	19.13%	20.52%	19.11%		21.24%	21.66%	21.27%	
Middle	20.50%	22.83%	20.47%		20.14%	23.88%	20.08%	
Richer	21.45%	15.10%	21.53%		20.57%	7.42%	20.75%	
Richest	22.43%	21.14%	22.44%		19.86%	3.11%	19.70%	
Place of residence:								
Urban	35.52%	41.08%	35.45%	0.075	33.29%	45.88%	33.02%	0.099
Rural	64.48%	58.92%	64.55%		66.71%	54.12%	66.98%	
Religion								
Muslim	14.28%	11.16%	14.33%	0.170	16.74%	5.46%	16.98%	0.003
Hindu	80.27%	84.12%	80.22%		78.08%	91.32%	77.90%	
Others	5.45%	4.71%	5.46%		5.18%	3.22%	5.13%	
Caste								
SC/ST	29.89%	39.81%	29.76%	0.005	30.58%	32.46%	30.52%	0.228
OBC	45.90%	38.19%	46.00%		46.17%	34.40%	46.45%	
Other caste/General	24.21%	22.00%	24.24%		23.25%	33.14%	23.03%	
Region of residence								
North	13.83%	6.12%	13.94%	0.000	14.15%	4.62%	14.38%	0.098
West	15.49%	10.96%	15.55%		15.73%	13.06%	15.81%	
South	23.27%	38.20%	23.07%		18.90%	31.67%	18.50%	
Northeast	3.46%	2.12%	3.48%		3.11%	1.50%	3.03%	
East	21.75%	22.09%	21.74%		21.43%	25.45%	21.39%	
Central	22.19%	20.51%	22.21%		26.69%	23.72%	26.87%	
Told someone or sought help from anyone (among those who experienced sexual violence)								
Yes	-	32.00%	-		-	28.67%	-	
No	-	68.00%	-		-	71.33%	-	
Person from whom the victim sought help (among those who experienced sexual violence)								
Own family	-	20.14%	-		-	15.74%	-	
Husband/partner family	-	6.20%	-		-	0.00%	-	
Current/former boyfriend	-	0.20%	-		-	0.13%	-	
Neighbour	-	1.66%	-		-	0.00%	-	
Social service organization	-	1.38%	-		-	6.05%	-	
Friend	-	6.56%	-		-	5.60%	-	
Police	-	1.45%	-		-	0.42%	-	
Religious leader	-	0.66%	-		-	0.00%	-	
Lawyer	-	0.11%	-		-	0.04%	-	
Doctor	-	0.00%	-		-	0.00%	-	
Others	-	0.68%	-		-	2.14%	-	
Relationship to perpetrator for first instance of sexual violence (among those who experienced sexual violence)								
Current or former boyfriend/husband	-	44.63%	-		-	1.68%	-	
Current or former boyfriend	-	5.90%	-		-	16.79%	-	
Father or step-father	-	3.07%	-		-	2.14%	-	
Brother or step-brother	-	2.60%	-		-	7.45%	-	
Other relatives	-	23.73%	-		-	27.16%	-	
In-laws	-	1.89%	-		-	0.98%	-	
Own friends or acquaintances	-	5.55%	-		-	13.31%	-	
Family friend	-	7.08%	-		-	14.31%	-	
Teacher	-	1.65%	-		-	4.61%	-	
Employer or someone at work	-	1.18%	-		-	0.001%	-	
Stranger	-	4.37%	-		-	5.60%	-	
Others	-	3.66%	-		-	5.95%	-	

### 3.1 Correlates of NMSV for complete sample (15–49 years)

From the ITA process, we identified eight themes that were associated with NMSV (Table 2). Results of the first round of lasso followed by ridge regression models generated four themes: Experiences of/Exposure to Violence, Geography, Economic Circumstances/Engagement/Empowerment, and Health Care Access/Use. The second iteration generated two additional themes: Maternal Health and Household Agency. The third round found two new themes – Knowledge of and Access to Sexual and Reproductive Health Services, and Sexual Behavior. We found no new variables in the fourth round, thus halting the iterative process. Two variables could not be categorized into any theme due to absence of related variables – a variable on nutrition/diet (occasional consumption of fruits), and a second variable on access to media (monthly visit to the cinemas/movie theatre), though these do suggest discretionary income.

**Table 2:** Identified predictors for corresponding themes from the iterative thematic analysis (ITA) for complete sample of women of age 15–49 years old.

Experiences of/Exposure to Violence	Geography	Health care access/use	Economic Circumstances/ Engagement/ Empowerment	Household agency	Maternal health	Sexual behaviour	Knowledge of and access to Sexual and Reproductive Health (SRH) Services
Has experienced violence during a pregnancy	State: Karnataka	Was told about side effects of family planning methods by health worker	Respondent works/is employed with someone outside of family	Husband alone usually decides on visits to family or relatives	During delivery, did not experience excessive bleeding	Total lifetime number of sex partners: one	Has not sought STI advice/treatment from: private hospital/clinic/private doctor
Has experienced physical violence, perpetrated by anyone other than husband	District: Buldana (state: Maharashtra)	Received two tetanus injections before pregnancy	Respondent alone usually decides what to do with money husband earns	Respondent alone decides on respondent's health care	During delivery, friend/relative assisted in childbirth	Is not married and no sex in last 30 days	Has not sought STI advice/treatment from: public government hospital
Timing of first instance of domestic violence (physical/sexual) in years after marriage: 0 years	Respondent's mother tongue: Bengali	Received Hepatitis B injection	Getting medical help for self: getting money needed for treatment is a big problem		Has had a pregnancy terminated	Had sex with most recent partner the last time: 100:130 days ago	Has not sought STI advice/treatment from any other sources
Timing of first instance of domestic violence (physical/sexual) in years after marriage: 2 years		Respondent's child has received the polio vaccine					Knows of some modern contraceptive method
Has physically hurt husband/partner when he was not hurting her		The time the respondent got any injection in last 12 months, the health worker took the syringe and needle from a new, unopened package					
Respondent's father beat her mother		During pregnancy, given or bought iron tablets/syrup					
Experienced either physical and/or sexual intimate partner violence		Has had a pregnancy terminated					
Is afraid of husband/partner most of the time		Person who usually decides on respondent's health care: respondent alone					
		Getting medical help for self: getting money needed for treatment is a big problem					
		Can access a condom if needed					

**Fig. 1:**
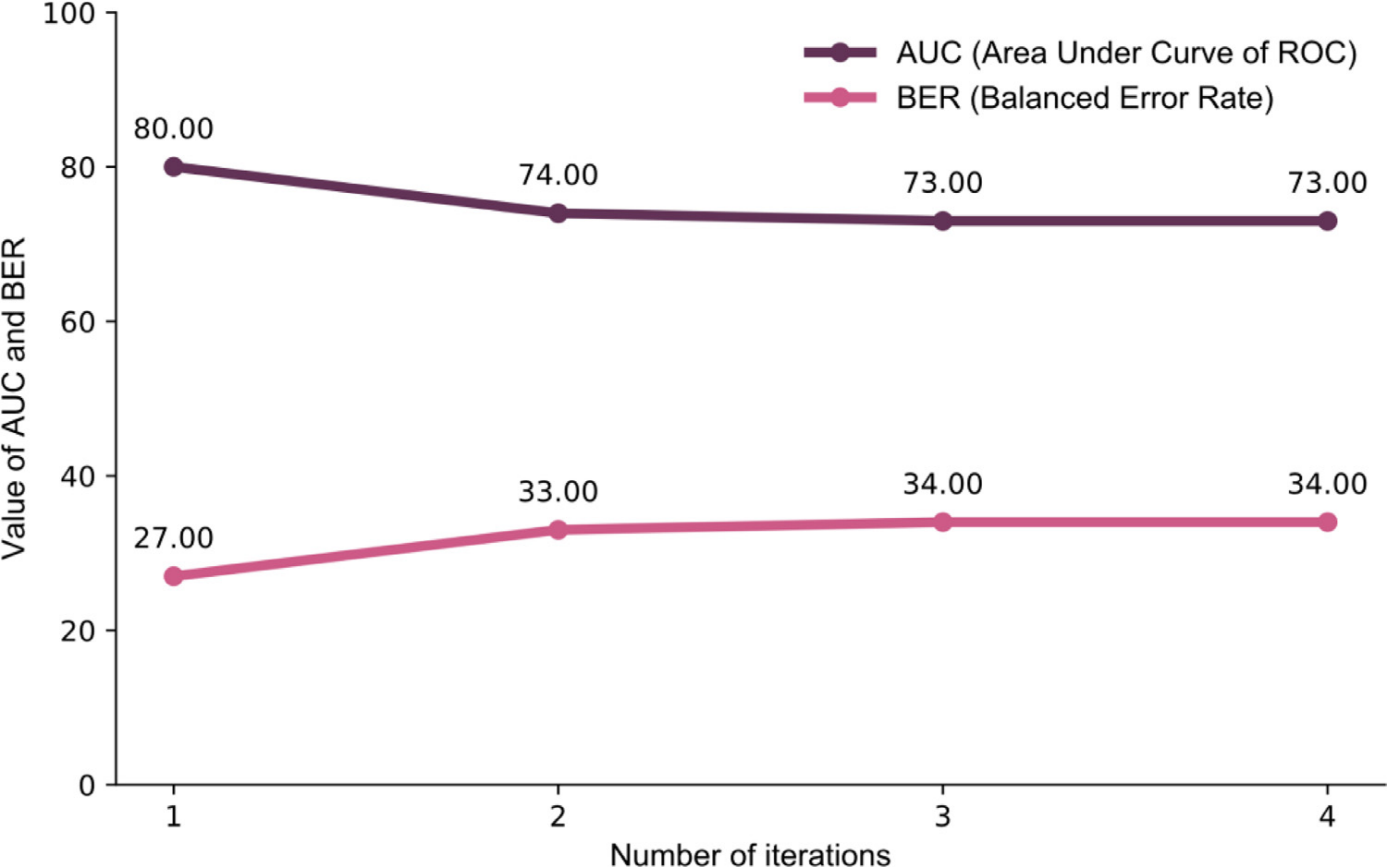
AUC and BER values for different rounds of the iterative models and iterative thematic analysis (ITA) process for complete sample. Findings indicate good reliability and low error for each of our iterative models 1–4.

The first and the final regularized regression models in the iterative categorization had AUC of 80% and 73% respectively, and error rates of 27% and 34% (Fig. 1).

The neural network model identified 20 features that were associated with NMSV, and had a coefficient value higher than the knee point of the coefficient curve (Table 3). The majority of these features were also identified in the iterative models and ITA process. Additionally, features related to many of the themes elicited in the above noted ITA process, specifically: Experiences of/Exposure to Violence, Economic Circumstances/Engagement/Empowerment, Knowledge of and Access to Sexual and Reproductive Health Services, but here we also found Access to Information or Media. The neural network model had AUC value of 79%.

**Table 3:** Identified predictors from neural network model for complete sample of women of age 15–49 years old.

**Neural network model variables**^1^**(coefficient value)**
Has experienced violence during a pregnancy (0.46)
Has experienced physical violence, perpetrated by anyone other than husband (0.36)
Has experienced physical and/or sexual intimate partner violence (0.29)
Has experienced emotional intimate partner violence (0.28)
Timing of first instance of domestic violence (physical/sexual) in years after marriage: 0 years (0.26)
Had sex with most recent partner the last time: over 299 days ago (0.23)
Is afraid of husband/partner most of the time (0.23)
Respondent's occupation: agricultural (0.22)
State: Uttar Pradesh (0.22)
Owns a house alone as well as jointly (0.21)
Has had no injections in the last 12 months (0.21)
Husband/partner's occupation: Services (0.21)
Respondent's father beat her mother (0.20)
Last source of contraceptive for users by type: government clinic/pharmacy (0.20)
Has received result from the last HIV test (0.20)
Frequency eats fish: daily (0.20)
Wealth index – urban and richer (fourth wealth index quintile) (0.19)
Respondent earns more money than husband/partner
Source of information about HIV/AIDS: Radio
Last contraceptive method discontinued in last 5 years: Withdrawal

1 predictors are those features that are above the knee point in coefficient curve; predictors presented in order of coefficient size (largest to smallest)

### 3.2 Correlates of NMSV for never married adolescent girls (15–19 years)

In replicating our approach with the subsample of never married girls, the first round of the ITA process identified 13 themes as correlates of NMSV (Table 4). We found that all themes identified in the overall sample of women were also in this subsample. Additional themes identified included: Attitudes Toward Marital Violence, Freedom of Movement or Mobility, Nutrition, Access to Information or Media, Tobacco and Alcohol Consumption, Menstrual Health and Hygiene, TB Knowledge and Behavior, Child Sex Preference, and Social Positioning (caste, education and rural/urban). We found no new variables in the second iteration, and the process of ITA thus ended. The AUC of the models in the two iterations were 86% and 77%. The models had BER of 30% and 40% respectively.

**Table 4:** Identified predictors for corresponding themes from the iterative thematic analysis (ITA) for never married adolescent sample (15–19 years).

Experiences of or exposure to violence, and attitudes toward spousal violence	Knowledge of and access to Sexual and Reproductive Health (SRH)	Freedom of movement or mobility	Economic circumstances, engagement and empowerment	Health care access and use	Nutrition	Access to information or media
Someone physically hurt respondent	Has heard about other STIs	Has been away for more than one month in last 12 months	Wealth index – rural: poorer	Getting medical help for self: having to take transport is a big problem	Frequency eats fried food: daily	Frequency of watching television: less than once a week
Respondent's father beat her mother	Source of information about AIDS: adult education programme	Usual resident or visitor: visitor	Wealth index: richest	Getting medical help for self: concern no provider is a big problem	Frequency takes milk or curd: never	Heard family planning on TV last few months: no
Beating justified if wife doesn't cook food properly	Source of information about AIDS: radio	Getting medical help for self: having to take transport is a big problem	Wealth index - urban: richest	Source for condoms: private: pharmacy/drugstore	Frequency eats fruits: never	Frequency of listening to radio: less than once a week
Beating justified if wife argues with husband	Source of information about AIDS: health workers	Has been away from home, other than parents’ home, for six months or more	Respondent works for family, others, self: for someone else	Source of FP: pharmacy/drugstore	Frequency takes aerated drinks: never	
Beating justified if wife argues with husband	Ways to avoid HIV/AIDS: use only new/sterilized needles	Has not been away from home, other than parents’ home, for six months or more		Visited a health facility or camp for self or children in last 3 months		
Beating justified if wife neglects the children	Ways to avoid HIV/AIDS: avoid sex with persons who have many partners			Type of health facility or camp visited for self or children in last 3 months: other		
Beating justified if wife refuses to have sex with husband	Does not know if people with HIV be allowed to work in the same office with people who do not have HIV			Type of health facility or camp visited for self or children in last 3 months: private or NGO		
Justifies domestic violence if wife is unfaithful	Does not know if HIV transmitted during delivery			Services/matters talked about in last 3 months: medical treatment for self		
	HIV not transmitted by breastfeeding			Service went for: medical treatment for self		
	People can get HIV/AIDS from blood products or blood transfusions			Blood pressure has been checked previously		
	Always using condoms during sex does not reduce risk of getting HIV			Never heard of oral rehydration		
	Having 1 sex partner only, who has no other partners can reduce risk of getting HIV					
	Would not buy vegetables from vendor with HIV					
	Would want HIV infection in family to remain secret					
	Can get HIV by sharing food with person who has aids					
	Place for HIV test: private: hospital/clinic/private doctor					
	Knows condoms					
	Knows IUD					
	Does not know foam/jelly					
	Does not know if they can get a condom					
	Knows a source for condoms					
	Source for condoms: ration shop					
	Source for condoms: private: pharmacy/drugstore					
	Source for condoms: ration or other shop or vending machine					
	Source of FP: pharmacy/drugstore					
	Has not heard family planning on TV last few months					
	Seen family planning message on a wall painting or hoarding					
	Knows that ovulatory cycle begins after period ends					

Tobacco and alcohol Consumption	Menstrual health and hygiene	Sexual behaviour	TB knowledge and behaviour	Gender parity - desire for girl/boy child	Social positioning - caste, education and rural/urban	
Someone smoked in respondent's home or presence, in last 30 days	Protection to prevent bloodstains: not cloth	Time since last sex (in days): 31+ days	Tuberculosis not spread by: food	Ideal number of girls: 2	Belong to other backward class	
Frequency drinks alcohol: less than once a week	Protection to prevent bloodstains: sanitary napkins	Has not had sex in last month	Tuberculosis spread by: sexual contact	Ideal number of boys: 2		
Uses tobacco		Reason for not having sex: other	Tuberculosis not spread by: touching a person with tb	Ideal number of children: 3+		

The neural network model for the adolescent girls sample identified variables related to Experiences of/Exposure to Violence and Knowledge of and Access to Sexual and Reproductive Health Services (Table 5). The model had AUC and BER of 74% and 34% respectively.

**Table 5:** Identified predictors from neural network model for adolescent sample (15–19 years).

**Neural network model variables**^1^**(coefficient value)**
Ways to avoid HIV/AIDS does not include use only new/sterilized needles (0.85)
Ways to avoid HIV/AIDS does not include avoiding sex with homosexuals (0.83)
Ways to avoid HIV/AIDS does not include avoiding sex with persons who inject drugs (0.82)
Someone physically hurt respondent (0.79)
Ways to avoid HIV/AIDS: don't know (0.78)
Ways to avoid HIV/AIDS does not include avoiding sex with sex workers (0.78)
Ways to avoid HIV/AIDS does not include avoiding sharing razors/blades (0.78)
Ways to avoid HIV/AIDS does not include limiting the number of sexual partners (0.77)
Ways to avoid HIV/AIDS does not include avoiding sex with persons who have many partners (0.77)
Ways to avoid HIV/AIDS does not include using condoms (0.75)
Ways to avoid HIV/AIDS does not include avoiding kissing (0.75)
Respondent's father beat her mother (0.75)

1 predictors are those features that are above the knee point in coefficient curve; predictors presented in order of coefficient size (largest to smallest)

## 4 Discussion

The current study confirms previous research in demonstrating that the strongest correlate of NMSV exposure is prior exposure to other forms of violence [27], [28], [29], [30]. specifically experiencing physical violence, marital violence, and witnessing violence against their mothers. Findings from our analysis with never married adolescent girls also found the above noted correlates seen for the full sample, and found that poorer, rural, and marginalized caste girls were more likely to report NMSV, the latter finding seen in prior demographic research using these data, [12] again validating our machine learning approach. These findings build on prior evidence indicating the validity of machine learning for identification of potential risk factors for gender-based violence indicators using large-scale survey data with a wide set of variables.

At the same time, use of this machine learning approach with iterative models and iterative thematic analysis (ITA) also elucidates novel variables associated with NMSV. For our total sample of 15-49 year olds, low sexual and reproductive health (SRH) knowledge and services was one of the strongest set of thematic predictors in this analysis, from both ITA methodology and neural network models. No/low sexual activity and NMSV were also associated with this sample, and may be tied to finding related to low SRH service utilization (e.g., no STI testing). In contrast, we find other types of health care access and use, decision-making control over healthcare utilization, and better maternal health indicators were associated with NMSV, as were income generation and control over household funds. Emerging from this picture is a women with economic and health autonomy but disconnection from sexual behaviour and SRH services. Sexual avoidance may be due to history of NMSV; prior research with women in India document this as a strategy for protecting oneself from sexual violence [31]. Of note, we also found this association in our never married adolescent subsample. However, given the cross-sectional nature of findings, we cannot presume which occurred first. Nonetheless, these findings suggest the value of supporting positive and healthy sexuality as part of sexual violence services and integration of SRH and sexual violence screening into other health services.

For our subsample of never married adolescents, findings were more complex. With regard to violence correlates, in addition to direct exposure to violence, girls reporting attitudes of acceptability toward marital violence were more likely to report NMSV. In terms of SRH knowledge and services, low knowledge about HIV/AIDS transmission and negative attitudes about people with HIV/AIDS were associated with NMSV, as were higher fertility preferences than that reported by Indian women as whole [12]. Health knowledge and health care access, media access, and menstrual hygiene access and use were also associated with NMSV, as were tobacco and alcohol use, the latter findings seen in prior research with adolescents from other country contexts [32], [33], [34], [35] but not previously identified in India. Research documents alcohol use as both a risk factor for sexual violence and a coping mechanism subsequent to such violence [35]. This pattern of good health knowledge outside of the area of HIV combined with more traditional and gender restrictive views regarding acceptability of marital violence, social exclusion of those living with HIV, and higher fertility preferences again suggest the value of SRH for these adolescent girls, with integration of gender equity norms and choices in SRH programs. There is particular need to address attitudes accepting of marital violence as these may render girls with a history of NMSV more vulnerable to such violence when married; prior research from India shows that such attitudes are associated with direct experiences of marital violence among women [36].

Additional correlates related to NMSV among never married adolescents included wealth and mobility, which support and extend prior epidemiologic analysis of these data [15]. We found that in addition to rural poor and backward caste associations with NMSV, richer and urban rich were also associated with NMSV, as was income generating. As with women overall, we see that among adolescent girls, those with economic access and agency are more likely to have experienced NMSV. Financially autonomous women and girls may have greater freedom to engage with society, which can also affect their exposure to NMSV. Alternatively, those who have experienced NMSV may strive for greater economic independence as a means of self-protection and security. For adolescent girls, findings related to greater mobility (e.g., greater time away from home, living in a home as a visitor) and NMSV, which correspond but do not perfectly align with prior study revealing the association between freedom of movement (i.e., ability to independently be in public spaces) and NMSV, [15] may be connected to this greater economic autonomy. These indications of temporary migration may be connected to girls working and earning, which again, may increase risk for NMSV exposure. It may also be the case that women with a history of NMSV are more likely to seek migratory and economic opportunities to escape ongoing sexual vulnerability, as most perpetrators of NMSV are individuals known to victims; again, the cross-sectional nature of these data impede assumptions related to causality. Temporary migration may also relate to educational opportunity for girls, but lack of educational correlates with NMSV suggests this not to be the case. Further, use of a household sample suggests respondents are not in school residence. Mobility should not heighten risk for NMSV, nor should NMSV affect such freedom. Freedom of movement and safety with migration, as well as safety at home, should be fundamental rights for all women and girls in India, but these data suggest this is not the case for many.

Study findings should be considered in light of certain study limitations. Data rely on self-report and, as already noted, under-reporting of NMSV is likely; relatedly reporting biases may exist in ways we cannot know from these data or analyses. For example, disclosure of violence to others may be higher in those willing to report violence in the survey, and thus, the prevalence of disclosure may be lower than what we find in the current analyses. Additionally, reporting may be indicative of more severe violence, which may explain higher than expected rates of disclosure in this study. Self-report data are subject to both recall bias as well as social desirability bias. Data are also five years old, but findings are unlikely to be different given no policy changes have occurred since then, including under COVID-19. Despite these limitations, these data are the only data on non-marital sexual violence available for a population-representative sample of women, offering important insight into the issue at a national level.

Additionally, while NFHS data have many variables, the survey is not focused on NMSV and thus does not have a comprehensive assessment of NMSV experiences, likely yielding further under-reporting and potentially having inadequate correlates for consideration. Measurement error may also be a concern, as there is limited information about potential data artifacts from this data set [1]. At the same time, for some correlates, variables were seen that go in the opposite direction. For example, among unmarried adolescents, both living away from parents’ home for six months or more AND not living away from parents’ home for six months or more were associated with NMSV. In such cases, thematic analysis proved particularly important. The former variable corresponded with more of the other variables under this theme, including being away from home for more than one month in the past 12 months and being a visitor in the current residence. Finally, data are cross-sectional, so causality cannot be assumed.

There is a need to apply machine learning to longitudinal data on this topic, allowing for consideration of causality. Unfortunately, such data are not available in India, and this analysis allows for some understanding of the issue with a nationally representative sample. Hence, we do feel the paper offers an important contribution to the literature, both in terms of the novel analytic approach, and because it does offer some novel findings previously unseen in prior study on the topic in India and with NFHS data. Novel findings include increased clarity on mobility as a risk (e.g., work for adults and greater time away from home for adolescents), and it connection with increased economic opportunity and autonomy.

Findings from this study demonstrate the validity and utility of machine learning with an iterative thematic analysis (ITA) to elucidate understanding of under-reported gendered phenomena such as NMSV in India. Findings correspond with prior epidemiologic and demographic research demonstrating that exposure to other forms of violence (e.g., physical violence, marital violence, witnessing of violence against mothers) is associated with NMSV [27], [28], [29], [30]. as is socioeconomic vulnerability [12]. Hence, this paper also contributes to the growing work documenting the utility and validity of applied machine learning combined with iterative thematic analysis (ITA) to examine social health issues such as violence.

This research also extends our understanding of NMSV in India by demonstrating that, among women and girls 15-49, those with economic agency (e.g., income generation, control over income) and health care knowledge and access are more likely to report NMSV, as are girls with high mobility and non-residence with family. These findings belie assumptions that the most socially vulnerable girls are at higher odds for sexual violence. While these agency indicators may be a consequence rather than a predictor of violence, prior research suggests that this is unlikely the case [31,37]. Future research needs to examine how and why more autonomous women and girls in India are at increased risk for NMSV. For example, if women with agency are more likely to be targeted, or if coercive sexual environments allow for increased violence, interventions will need to target norms regarding respect for women and safety in public spaces to reduce NMSV experiences. Findings related to poor HIV knowledge, low use of STI services, and no sexual behavior additionally suggest the importance of SRH services. Such services for adolescents should integrate gender equity norms to reduce attitudes of acceptability toward marital violence, given its association with NMSV, and to reduce vulnerability of girls who experienced NMSV to risk for marital violence, a finding also suggested in this study. In sum, this research demonstrates the utility of these exploratory findings using machine learning and ITA for future hypothesis driven analysis and more responsive policy and programs.

## Funding

This work was funded by the Bill and Melinda Gates Foundation, Grant OPP1179208 (PI: Raj).

## Data Sharing

These data are publicly accessible from the Demographic and Health Survey website. The code required to replicate the analysis would be available upon request to the authors.

## Contributors

Anita Raj led the development of the research concept and writing of the manuscript. She conducted the review of the literature. Nabamallika Dehingia conducted data analyses and supported the literature review. She drafted the methods and results; drafted all tables and provided substantive revisions to the document. Abhishek Singh procured the data, supported drafting of the methods and provided substantive revisions to the document. Julian McAuley led development of the machine learning analytic approach and guided data analysis. He provided substantive review of the document. Lotus McDougal co-led conceptualization of the concept and substantively contributed to all sections of the paper. All of the authors revised the manuscript and approved the final version before submission.

## Declaration of Competing Interest

All the authors report support from the Bill and Melinda Gates foundation during the conduct of the study.

## Appendix Supplementary materials

Image, application 1
