# Supplementary material for: Machine learning analysis of non-marital sexual violence in India
Source: eClinicalMedicine. 2021 Aug 1;39:101046. doi: 10.1016/j.eclinm.2021.101046 (PMC8350001; doi:10.1016/j.eclinm.2021.101046)
Supplement: Supplementary file 1 [file mmc1.docx]

Appendix A: Details on machine learning models

Data pre-processing. The Demographic and Health Survey (DHS) dataset used in the study was first processed to make it suitable for analysis. Two researchers in the team reviewed each variable in the dataset to identify repetitions and redundant variables, as well as survey design and structure variables, which were excluded from the analysis. For example, variables related to date of interview, respondent IDs etc. were removed, as they did not describe characteristics of the respondent herself. DHS also included multiple variables for the same construct. Age, for example, was captured by multiple variables (continuous age variable and categorical variables with different age categories). Variables were dropped to ensure that each construct was captured by a single variable in the dataset. Once the unnecessary variables were dropped, the researchers identified continuous variables that needed to be categorized, or converted to categorical variables. These categorization decisions were based on how variables were categorized in prior research, to ensure consistency of interpretations with existing literature.

Below, we describe the two machine learning regularized models used in the study, and how they differ from traditional regression models.

Traditional Regression. Traditional logistic regression with a binary response is denoted by the equation:

$$P\left( y=1 \right)= \frac{1}{1+ e^{-\theta\beta}}$$

We obtain regression coefficients by maximizing the log-likelihood function:

$$l_{\theta}\left( y | X \right)= \sum_{i} -log(1+ e^{{-X}_{i}\theta})+ \sum_{y_{i}=0} -X_{i}\theta$$

Where X is the vector of features or variables and θ is the column vector of the regression coefficients. However, in cases involving high dimensionality, or large number of features, the ordinary logistic regression has a few problems: multicollinearity, and over-fitting, and computational difficulties.

Lasso. To address concerns posed by the presence of large number of features in a dataset is regularization. Regularization is a form of regression that imposes a penalty on the size of logistic regression coefficients, trying to shrink them towards zero. Regularized estimators are thus restricted maximum likelihood estimators (MLE), since they maximize the likelihood function subject to restrictions on the logistic regression parameters.

To develop parsimonious ridge and neural network models, with fewer and more relevant features, we used lasso. Lasso uses an L-1 penality for both variable selection and shrinkage, and when the λ is sufficiently large, it can force some of the coefficient estimates to be exactly equal to zero, giving us models with lesser number of predictors/features. The log-likelihood function for lasso takes the form:

$$l_{\theta}\left( y | X \right)= \sum_{i} -log(1+ e^{{-X}_{i}\theta})+ \sum_{y_{i}=0} -X_{i}\theta- \lambda|\theta|$$

The difference between the traditional regression and lasso is the last term: $\boldsymbol{\lambda|\theta|}$**.** This term is the regularizer and is used to optimize the log-likelihood function. Simply put, this allows the model to carry out multiple iterations for the log-likelihood function to find the best values for all the betas (coefficients) in the equation, while mitigating overfitting and bias.

Ridge. Ridge or L-2 regularized logistic regression is also obtained by maximizing the log-likelihood function with a penalized parameter applied to all the coefficients except the intercept, resulting in the following constrained maximization equation:

$$l_{\theta}\left( y | X \right)= \sum_{i} -log(1+ e^{{-X}_{i}\theta})+ \sum_{y_{i}=0} -X_{i}\theta- \lambda{|\left| \theta\right||}_{2}^{2}$$

Where λ is the tuning parameter for the L-2 regularized model. The larger the λ, the stronger its influence is, and the smaller are the parameter estimates. When λ = 0 the solution is the ordinary MLE, whereas if λ→∞, the θ all tend to 0. Different approaches to choose the value of λ have been described in existing literature. The tuning parameter for ridge, λ, was selected using k-fold cross validation, using the method described above for lasso.
